# A Facile Electrode Modification Approach Based on Metal-Free Carbonaceous Carbon Black/Carbon Nanofibers for Electrochemical Sensing of Bisphenol A in Food

**DOI:** 10.3390/foods14020314

**Published:** 2025-01-18

**Authors:** Jin Wang, Zhen Yang, Shuanghuan Gu, Mingfei Pan, Longhua Xu

**Affiliations:** 1 College of Food Science and Engineering, Shandong Agricultural University, Tai’an 271018, China; wj767218291@163.com (J.W.); u16112806@163.com (Z.Y.); 17662631370@163.com (S.G.); 2 College of Food Science and Engineering, Tianjin University of Science and Technology, Tian’jin 300457, China

**Keywords:** Bisphenol A, carbon nanofibers, carbon black, electrochemical sensor

## Abstract

Bisphenol A (BPA) is a typical environmental estrogen that is distributed worldwide and has the potential to pose a hazard to the ecological environment and human health. The development of an efficient and sensitive sensing strategy for the monitoring of BPA residues is of paramount importance. A novel electrochemical sensor based on carbon black and carbon nanofibers composite (CB/f-CNF)-assisted signal amplification has been successfully constructed for the amperometric detection of BPA in foods. Herein, the hybrid CB/f-CNF was prepared using a simple one-step ultrasonication method, and exhibited good electron transfer capability and excellent catalytic properties, which can be attributed to the large surface area of carbon black and the strong enhancement of the conductivity and porosity of carbon nanofibers, which promote a faster electron transfer process on the electrode surface. Under the optimized conditions, the proposed CB/f-CNF/GCE sensor exhibited a wide linear response range (0.4–50.0 × 10^−6^ mol/L) with a low limit of detection of 5.9 × 10^−8^ mol/L for BPA quantification. Recovery tests were conducted on canned peaches and boxed milk, yielding satisfactory recoveries of 86.0–102.6%. Furthermore, the developed method was employed for the rapid and sensitive detection of BPA in canned meat and packaged milk, demonstrating comparable accuracy to the HPLC method. This work presents an efficient signal amplification strategy through the utilization of carbon/carbon nanocomposite sensitization technology.

## 1. Introduction

Bisphenol A (BPA), as a chemical monomer for the preparation of polycarbonate plastic and epoxy resin, is widely used in the manufacture of plastic food containers and food cans [1,2]. BPA usually remains in packaging materials after polymerization. With the change in storage conditions such as heat treatment and long-term storage, BPA will be accelerated to migrate from food containers and packaging materials to food [3]. Human exposure to BPA will increase the risk of reproductive dysfunction, cardiovascular disease, diabetes, and obesity [4]. Considering the intake of BPA from food and related health problems, a specific migration limit of 0.05 mg BPA per kg of food from food contact materials was applied under Regulation (EU) 2018/213 [5]. Therefore, it is significant to establish a high sensitivity and rapid response method for detecting BPA residues in food and the environment and reducing exposure hazards.

In recent years, various analytical techniques have been applied for the determination of BPA, including high-performance liquid chromatography (HPLC) [6], surface-enhanced Raman scattering (SERS) [7], liquid chromatography–mass spectrometry [8], and fluorescence sensors [9]. They have the advantages of a wide linear range and good selectivity. In addition to the aforementioned advantages, electrochemical sensors have garnered increasing attention for their potential in rapid analyte detection, owing to their simplicity, rapid response, high sensitivity, and ease of miniaturization.

Electrochemical sensing is a method to quickly detect the target by converting the chemical signals generated from the oxidation–reduction reaction of substances into electrical signals. Thinking in term of structure, BPA as an electrochemically active species could be directly detected by electrochemical sensors, because it contains two phenolic hydroxyl groups that can be oxidized to quinone by losing two electrons to generate an electrochemical signal. While in fact, a poor response usually occurs on the traditional unmodified electrodes because the oxidation process of BPA is irreversible and requires overpotential, as well as the formed oxidation products adsorbing on the electrode, causing contamination, which results in a low detection sensitivity. In order to solve the above problem, various attempts have been made to modify electrodes by employing many materials such as carbon nanotubes [10], quantum dots [11], metal nanoparticles [12], and so on.

Carbon-based nanomaterials show great application potential in electrochemical sensors because of their excellent catalytic performance, strong analyte adsorption capacity, wide potential window, and other advantages. Carbon nanofibers (CNFs), as an important member of the carbon materials, have become an exceptional candidate for electrode materials and immobilization substrates owing to their low cost, large specific surface area, excellent electrical conductivity, and easy functionalization [13,14,15]. Recently, researchers have been studying carbon nanomaterials such as graphite, graphene, carbon nitride, and carbon nanotubes (CNTs) and analyzing their use in electrochemistry to improve electrochemical peak performance. CNF is a carbon nanomaterial similar to carbon nanotubes, with unique mechanical and electrical properties. Moreover, the outer wall of CNFs has abundant edge active sites and low order, which enhances electron transfer, so the electrochemical activity of CNFs is significantly higher than that of CNTs [16]. Structurally, different from carbon nanotubes (CNTs), CNFs are formed by the stacking of graphene sheets of varying shapes, and there are more exposed edges sites on the outer walls of CNFs than CNTs, which can facilitate the electron transfer of electroactive analytes [17]. In addition, surface functionalization on CNFs is much easier than on CNTs by chemical oxidation to introduce the carboxyl and carbonyl polar functional groups, enhancing the dispersion as well as the reactivity of CNFs, which makes it easier to enrich analyte molecules to increase the electrochemical sensitivity. Recently, Zhang and his co-workers used the in situ reduction method to fabricate the Au/CNF composite for the electrochemical sensing of metronidazole [18], validating the role of CNFs as immobilization substrates of Au nanoparticles in synergistic sensitization for electrochemical analysis.

Carbon black (CB) has the characteristics of high electrocatalytic activity, cost-effective, stable dispersity, and no need for pretreatment [19,20,21]; therefore, it has been widely used in lithium-ion batteries [22], fuel cells [23], supercapacitors [24], and photocatalysis [25]. More attractively, CB exhibits excellent electrical conductivity and the possibility of facile functionalization and possesses numerous defective sites and fast electron transfer kinetics [26], which make it a promising electrode material for sensors. For instance, Dos Santos prepared a CB-modified glassy carbon electrode to detect the stimulant selegiline by adsorptive stripping voltammetry, which demonstrated that the introduction of CB nanoparticles endowed the glassy carbon electrode with high sensitivity for the analyte by strengthening the electrical conductivity [27]. It is worth noting that the electrochemical performance of CBs strongly depends on the availability of CB nanoparticles homogeneously and finely stabilized on the electrode. However, in real applications, due to the existence of van der Waals forces between the particles, CB is prone to form aggregates and then form agglomerates [28]. Hence, to seek an appropriate substrate to improve the dispersion of CB is still a key issue to be solved, and fortunately CNFs offer an invigorating alternative.

In this work, a nanocomposite material based on carbon black and functional carbon nanofibers (CB/f-CNF) was synthesized by a simple hybrid ultrasonic method. The CB/f-CNF with excellent conductivity and an outstanding catalytic performance was used to decorate the glassy carbon electrode (GCE), and a modified electrode named as CB/f-CNF/GCE was obtained. A novel electrochemical sensor based on CB/f-CNF/GCE was constructed and its performance for sensing BPA was further evaluated by cyclic voltammetry (CV) and differential pulse voltammetry (DPV). The accuracy and practical application of the CB/f-CNF/GCE sensor were also verified by recovery tests for canned peach and milk samples, and finally the proposed sensors were successfully employed for the detection of BPA residues in canned yellow peach, canned dace with black bean, and milk samples. To our best knowledge, this is the first study to develop a metal-free carbonaceous nanocomposite-based electrochemical sensing platform for the detection of BPA in foods.

## 2. Materials and Methods

### 2.1. Materials and Reagents

The canned peach in glass jars, canned yellow peach in plastic boxes, canned dace with black bean in metal cans, and milk, packaged in both paper cartons and plastic bags, were purchased from a local supermarket (Tai’an, China).

CNFs (>95%) and CB were bought from Nanjing XFNANO Materials (Nanjing, China). BPA and NaOH were purchased from Macklin (Shanghai, China). 4,4-dihydroxydiphenylmethane (BPF, >99%) was purchased from Aladdin Chemical Co., Ltd. (Shanghai, China). Hydroquinone (HQ), Catechol (CT), and Resorcinol (RC) were bought from Tianjin Kemiou (Tianjin, China). Bis(4-hydroxyphenyl) methylethylmethane (BPB, >99.5%) was purchased from Dr Ehrensorfer GmbH (Augsburg, Germany). Glacial acetic acid, phosphate, boric acid, nitric acid, sulfuric acid, n-hexane, acetonitrile, KCl, K_3_[Fe(CN)_6_], and K_4_[Fe(CN)_6_] were supplied by Tianjin Kaitong (Tianjin, China). The double-distilled water (DDW) obtained by the Millipore water purification system had a resistivity of 18.2 MΩ·cm^−1^.

The Britton–Robinson (BR) buffer solution used in this study consists of a mixture of 40 mM H_3_PO_4_, 40 mM CH_3_COOH, and 40 mM H_3_BO_3_, which was titrated to pH 7.0 with 0.2 M NaOH. We prepared a probe solution by combining 2.0 mmol·L_−1_ K_3_[Fe(CN)_6_], 2.0 mmol·L^−1^ K_4_[Fe(CN)_6_], and 0.2 mol·L^−1^ KCl in a BP buffer solution at pH 7.0.

### 2.2. Instruments and Apparatus

Powder X-ray diffraction (XRD) patterns were acquired using a Bruker D8 Advance instrument (Karlsruhe, Germany). Scanning electron microscopy (SEM) images were analyzed in an FE-SEM (S4800, HITACHI, Tokyo, Japan). Transmission electron microscopy (TEM) images were acquired using a JEM-1400 transmission electron microscope (JEOL, Tokyo, Japan). The Raman spectrum was measured with a DXR2xi microscopic Raman imaging spectrometer (Waltham, MA, USA). We used a glass carbon electrode (6 mm) saturated calomel electrode and a platinum wire electrode (Xianren Instrument Co., Ltd., Shanghai, China)

### 2.3. Construction of CB/f-CNF/GCE Sensor

**Figure 1:**
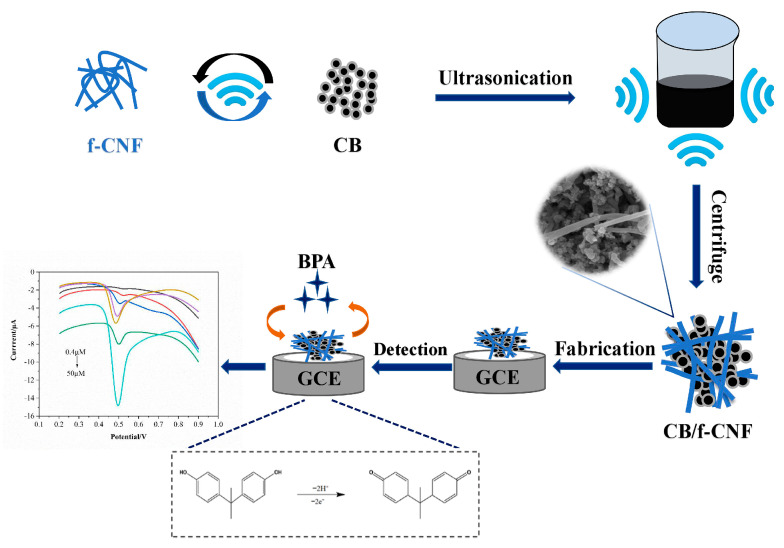
Schematic illustration of the construction of the CB/f-CNF/GCE sensor. Note: Black line, red line, blue line, purple line, yellow line, green line and cyan line are the DPV curves of CB/f-CNF/GCE in bisphenol A solution of 0.4 μM, 1 μM, 2 μM, 6 μM, 10 μM, 20 μM and 50 μM, respectively.

Prior to modification, the glassy carbon electrode (GCE) was polished to a mirror finish using 0.3 μm and 0.05 μm alumina slurry, followed by ultrasonic rinsing with deionized distilled water (DDW), ethanol, and DDW for 3 min each. The electrochemical behavior was characterized by a ΔE_p_ value of ≤ 110 mV. Firstly, the f-CNF was prepared by the acid treatment in a typical procedure, 1.0 g CNF was added to 40.0 mL HNO_3_/H_2_SO_4_ (1:3) solution and stirred continuously at 50 °C for 1 h, in order to attach the hydrophilic oxygen-containing functional groups to promote the self-dispersion and loading of the nanoparticles. Then, the f-CNF product was obtained after being rinsed with DDW until the pH reached about 7.0, and dried in a vacuum oven at 60 °C for 12 h. After that, a uniform suspension of CB/f-CNF composite was formed by mixing 2.5 mg CB and 2.5 mg CNFs in 2.0 mL DDW under ultrasound for 1 h. Finally, the modified CB/f-CNF/GCE sensor was fabricated using 8.0 μL of dispersion liquid dropped onto the surface of the GCE and dried at room temperature. The construction process of the CB/f-CNF/GCE sensor is illustrated in Figure 1.

### 2.4. Electrochemical Sensing of BPA

All electrochemical measurements were conducted using an electrochemical workstation (CHI650E, Shanghai, China) equipped with a standard three-electrode system. The working electrode was either a bare glassy carbon electrode (GCE) or a modified CB/f-CNF/GCE, with a platinum sheet acting as the counter electrode and a saturated calomel electrode (SCE) serving as the reference electrode.

The CB/f-CNF/GCE was immersed in a voltammetric cell containing different concentrations of BPA solution (BR, pH 7.0). The differential DPV scans were run (+0.2 V to +0.9 V) to record the oxidation peak current of BPA. The other conditions of DPV included an amplitude of 50 mV, a pulse width of 50 mV, a pulse period of 0.5 s, and a quiet time of 2 s. CV curves in the [Fe (CN)_6_]^3−/4−^ redox solution for different modified electrodes were performed at potentials in the range of −0.2 V~+0.6 V at a scan rate of 50 mV s^−1^. CV measurements of BPA were performed for +0.2 V~+1.0 V.

### 2.5. Preparation of Real Samples

The canned peach, canned dace, and milk packed with different materials were selected as real samples and prepared according to our reported method [29]. For canned dace, 5 g of the homogenized sample was extracted with a mixture solution of acetonitrile (10 mL) and hexane (10 mL) under vigorous shaking for 2 min. After centrifugal separation, the acetonitrile phase was transferred into a 25 mL polypropylene centrifuge tube. Another 10 mL of fresh acetonitrile was added to the solid residue and hexane for secondary extraction. The combined acetonitrile extract was filtered through a 0.22 μm membrane, evaporated to dryness at 50 °C. For canned fruit and boxed drinks, the above procedure was also adopted but without hexane. For milk, the pretreatment process was like that of canned meat products: 5 g of the homogenized sample was extracted with a solution of acetonitrile (5 mL) and hexane (5 mL). Another 5 mL of acetonitrile was used for secondary extraction, and the combined acetonitrile was filtered and evaporated to dryness. But the final extract was re-dissolved with 2 mL of BR buffer (pH = 7) or methanol solution, respectively, for further electrochemical or HPLC analysis.

### 2.6. Method Validation and Addition Recovery

After the sample was processed by the above method, the HPLC method [29] was compared with the CB/f-CNF/GCE sensor to detect the BPA content in the actual sample. The specific parameters of HPLC are shown in the Supplementary Materials, and three parallel experiments were performed to calculate the error.

In addition, to assess the accuracy of the CB/f-CNF/GCE sensor, recovery experiments were conducted by adding 0.10 mL of BPA standard solution at three concentrations into 5.00 g of homogenized canned peach in glass jars or milk in paper cartons. After overnight incubation at room temperature, the samples were treated with the same process, and the contents of BPA were determined with the proposed sensor.

## 3. Results and Discussion

### 3.1. Morphology and Structure Characterization

**Figure 2:**
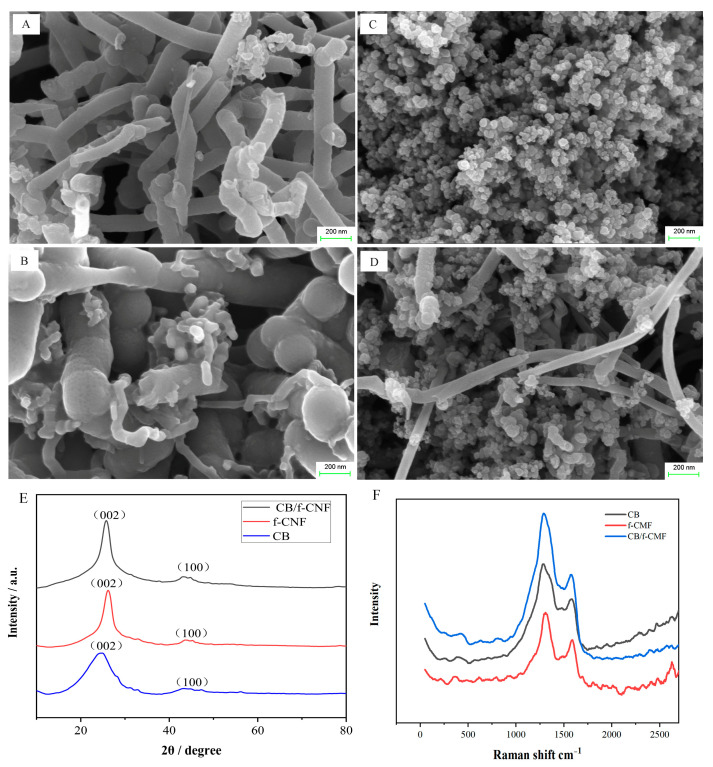
SEM images of CNF (**A**), f-CNF (**B**), CB (**C**), and CB/f-CNF (**D**). XRD patterns for the as-synthesized CB, f-CNF, and CB/f-CNF (**E**); Raman spectra of carbon black, f-CNF, and CB/f-CNF composite (**F**).

The surface morphologies of CNF, f-CNF, CB, and f-CNF/CB composites were examined using scanning electron microscopy (SEM), as shown in Figure 1. CNF exhibits a typical one-dimensional nanostructure (Figure 2A) with irregular arrangement; after acid treatment, f-CNF (Figure 2B) obtains a rougher surface compared to untreated CNF. The CB sample (Figure 2C) is composed of many sphere-like carbon nanoparticles which remain in close contact with each other throughout the point-to-point connection process and form a pearl chain of carbon conductive network structure, thus improving the effective electrical conductivity of the nanocomposite [30,31]. As shown in Figure 2D, CB nanoparticles are dispersed in the carbon conductive network structure of f-CNF, demonstrating the successful synthesis of CB/f-CNF. The structure and performance advantages of f-CNF and carbon nanoparticles can be fully exploited in CB/f-CNF. f-CNF possesses a higher charge transfer efficiency due to its hollow nanostructure, while CB has a shorter charge transfer path due to its chain-like network structure. The extensive point-to-point connections between f-CNF and CB facilitate the formation of a layered carbon conductive network, with both long-range and short-range conductive pathways. This is consistent with the TEM images of the CB/f-CNF composite shown in Figure S1. The interconnected carbon conductive network of CB/f-CNF provides more efficient ion and electron transfer channels, as well as a high electrochemically active surface area. These features enhance the conductivity and adsorption capacity of the CB/f-CNF composites [32,33].

The crystalline structure of the CB, f-CNF, and CB/f-CNFs was identified by XRD and their diffraction patterns are shown in Figure 2E. The characteristic diffraction peak of the (002) crystallographic planes of graphite crystallites appear in all three carbon-based materials, located at 2θ = 24.5° for CB, 2θ = 26.2° for f-CNF, and 2θ = 25.8° for CB/f-CNFs, respectively [34,35]. Compared to pure f-CNF, the peak of CB/f-CNFs shifts to a lower angle and the full width at half maximum increases, which suggests a somewhat reduced degree of graphitization in the composite materials due to the CB doping. In addition, the broad peaks with low intensity at around 43–44° (2θ) reveal the presence of another typical (100) plane of graphite crystallites in the three carbon materials.

Raman spectrometry was also employed to investigate the structural information of CB, f-CNF, and CB/f-CNF. As displayed in Figure 2F, the two dominant peaks at around 1300 cm^−1^ and 1585 cm^−1^ allocated for D and G graphite bands are present in all of the tested materials. Among them, for the pristine f-CNF, the corresponding peaks appear at 1308 and 1591 cm^−1^, while for CB/f-CNF, the peaks of D and G bands were detected at 1291 and 1576 cm^−1^, which are downshifted by 17 and 15 cm^−1^, respectively, when compared to f-CNF, which can be attributed to the introduction of CB in the composite material [36,37,38].

### 3.2. Electrochemical Characterization of CB/f-CNF/GCE

**Figure 3:**
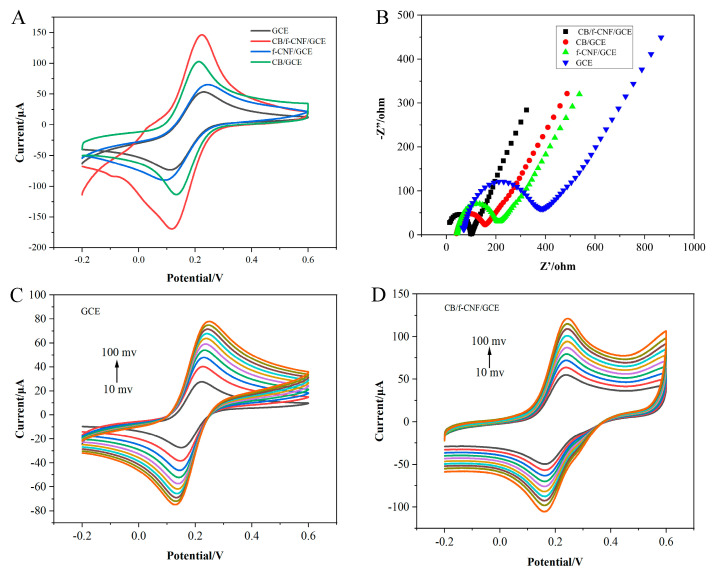
The CV (**A**) and EIS (**B**) in a [Fe(CN)6]^3−/4−^ redox probe solution response of GCE, CB/GCE, f-CNF/GCE, and CB/f-CNF/GCE. CV responses for GCE (**C**) and (**D**) The CB/f-CNF/GCE was analyzed at different scan rates, ranging from 10 to 100 mV s^−1^, in a 2.0 mmol·L^−1^ [Fe(CN)6]^3/4−^ solution. Note: Different lines from top to bottom are the CV curves of GCE and CB/f-CNF/GCE under the sweep speed of 10, 20, 30, 40, 50, 60, 70, 80, 90, 100 mv respectively.

Cyclic voltammetry was conducted to reflect the electrochemical performance of the different modified electrodes. In Figure 3A, well reversible redox peaks of [Fe(CN)_6_]^3/4−^ for all electrodes can be observed, and as expected, CB/f-CNF/GCE had the maximum peak current, followed by CB/GCE (89.0 μA), f-CNF/GCE (74.0 μA), and the minimum value of bare GCE (61.0 μA). In detail, the peak current of the bare GCE was accompanied by a potential difference (ΔEp) of 108 mV. After modification with CB, the ΔEp decreased to 102 mV, and the current increased, which can be attributed to the enhanced catalytic properties of CB. When the GCE was coated with f-CNF, the peak current increased slightly owing to the outstanding electrical conductivity of f-CNF. The current measured for the CB/f-CNF/GCE was significantly higher than that of the CB/GCE and f-CNF/GCE, ascribed to the synergistic electrochemical effect between CB and f-CNF.

Electrochemical impedance spectroscopy (EIS) was employed to investigate the interfacial properties of the different electrodes in a 2.0 mM [Fe(CN)6]^3/4−^ solution. The diameter of the semicircle represented the charge-transfer resistance (Rct) of the modified electrodes [39]. As shown in Figure 3B, the Rct of the bare GCE was 216.20 Ω. After the GCE was covered with CB (CB/GCE), the Rct changed to 121.37 Ω, attributed to the excellent electrocatalytic activity of CB. The Rct of the f-CNF/GCE was 160.78 Ω. A higher Rct value of the f-CNF modified electrode was observed due to the oxygen-containing functional groups on the surface of the f-CNF [40]. Fortunately, the CB/f-CNF/GCE exhibited a low charge transfer resistance (Rct) of 98.46 Ω. The electronic conductivity of the CB/f-CNF composites was significantly enhanced by the integration of highly conductive CB particles with defect-rich f-CNF surfaces, thereby confirming the synergistic effect between CB and f-CNF. These results suggest that CB/f-CNF composites are ideal materials for the fabrication of electrochemical sensors for BPA detection.

The electroactive surface area of the electrode is an important characteristic of electrochemical sensors. Figure 3C,D show CV curves for bare GCE and CB/f-CNF/GCE in [Fe(CN)_6_]^3−^/[Fe(CN)_6_]^4−^ redox probes at different scanning rates (10–100 mV s^−1^), and the electrochemical effective area of bare GCE and CB/f-CNF/GCE is calculated and compared by the Randles–Sevcik equation [41]: I_P_ = 2.69 × 105 × n^3/2^ × C_0_ × A × D_0_^1/2^ × v^1/2^, where I_P_ is the peak current (μA), n is the number of electron transfers, C_0_ denotes the concentration of the redox probe solution (mol·cm^−3^), A is the electroactive area (cm^2^), v indicates the scanning rate (V s^−1^), and the diffusion coefficient of the probe solution of D_0_ is 7.60 × 10^−6^ (cm^2^ s^−1^). In this study, the electroactive surface area of GCE and CB/f-CNF/GCE was calculated by the Randles–Sevcik formula to be 0.0836 cm^2^ and 0.164 cm^2^, respectively (Figure S2A,B), which is around two times that of the bare electrode, indicating that the CB/f-CNF composite material can increase the electrochemical effective area of the electrode and accelerate the electron transfer between the electrode and the redox probe, so the CB/f-CNF composite is an ideal nanomaterial that can be used to build sensors.

### 3.3. Electrochemical Response of CB/f-CNF/GCE Sensors Toward BPA

**Figure 4:**
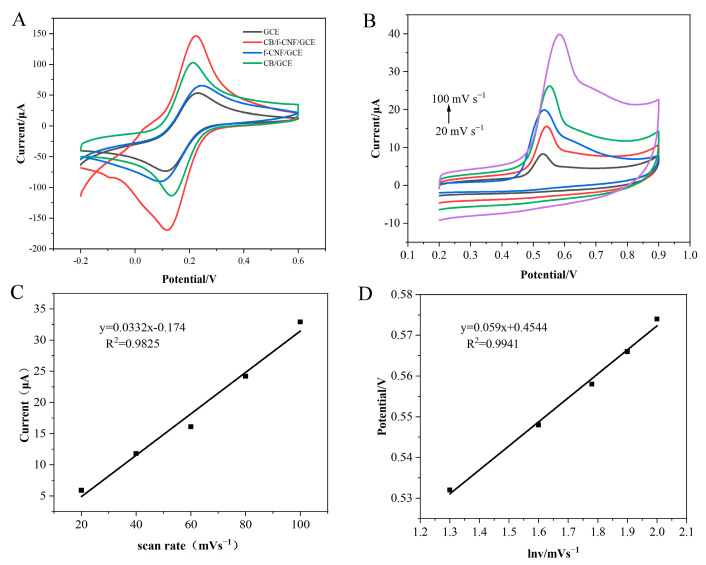
(**A**) CV curves of bare GCE, CB/GCE, f-CNF/GCE and CB/f-CNF/GCE in the BR containing 50 μmol L^−1^ BPA and CV curves of CB/f-CNF/GCE in a BPA-free BR solution (blank). (**B**) CV curves of CB/f-CNF/GCE in the BR containing 20 μmol L^−1^ BPA at various scan rates: 20, 40, 60, 80, and 100 mV s^−1^. (**C**) The linear relationship of the BPA oxidation peak currents versus the scan rates. (**D**) The relationship between the BPA oxidation peak potentials and the natural logarithm of scan rates. Note: The black, red, blue, green and purple lines are the CV curves of CB/f-CNF/GCE in BR containing 20 μmol L^−1^ BPA at: 20, 40, 60, 80 and 100 mV s^−1^ scanning rates, respectively.

To demonstrate the benefits of using the CB/f-CNF composite as the basis for the sensor, the electrochemical response CV measurement is a very convenient and effective electrochemical method for observing electron transport processes. In this experiment, CV was used to study the electrochemical response of BPA on different modified electrodes. As shown in Figure 4A, due to the poor sensitivity of the bare GCE, no peak current was detected on the bare GCE. After modification with CB, the peak current was about 9.80 μA, with the peak potential (Ep) of +0.783 V owing to the excellent conductivity of CB, and no reduction peak was observed during the reverse scan. The results show that the oxidation reaction of BPA is a completely irreversible process. The Ep of f-CNF/GCE was +0.758 V and the current was 3.99 μA, which may be attributed to the electrocatalytic ability of the f-CNF, while its large specific surface area and strong adsorption capacity are improved. The f-CNF/GCE increased the enrichment efficiency of BPA, thus promoting the catalytic oxidation of BPA. This may be attributed to the activation of the edges of the acid-functionalized CNF, which makes it easy to functionalize, thus introducing carboxyl and carbonyl polar functional groups, enhancing the dispersibility and compatibility of CNF, increasing its specific surface area, making it easier to adsorb BPA and improving its electrochemical performance [42]. For CB/f-CNF/GCE, Ep negatively moves to +0.766 V, the current increases to 19.30 μA, the background current is low, and the peak shape is narrow, which further demonstrates the synergistic effect between CB and f-CNF, and effectively improves the electrocatalytic effect of the electrode on BPA oxidation.

To investigate the oxidation process of BPA on CB/f-CNF/GCE, CV curves of the CB/f-CNF/GCE were determined in BR solution containing 50 μmol L^−1^ BPA at different scanning rates varied from 20 to 100 mV s^−1^ (Figure 4B). As the scanning rate increases, both the oxidation peak potential and current of BPA gradually increase. A linear relationship between the oxidation current and scanning rate is observed, expressed by the equation I_p_ = 0.0332v − 0.174 (R^2^ = 0.9825). The result demonstrated that the oxidation reaction of BPA was an adsorption-controlled process [20,43]. In addition, there is also a linear relationship between the oxidation peak potential of BPA and lnν, according to the Laviron equation: Epa = E^0^ + (RT/a n F) ln(RT k_s_/a n F)lnV, where R is the general gas constant, T is 298 K, α is the charge transfer coefficient of the oxidation reaction (0.5), F is the Faraday constant, n is the number of electrons in the electrode reaction, and the calculated electron transfer number is 2. Therefore, electrochemical oxidation of BPA in CB/f-CNF/GCE is a two-electron and proton transfer reaction [44].

### 3.4. Optimization of Test Conditions

In order to obtain the best performance of BPA detection, the experimental conditions such as the volume of CB/f-CNF dispersion and the pH value of the electrolyte solution were optimized in a 50 μM BPA solution in BR. As shown in Figure S3A, with the volume of CB/f-CNF dispersion increased from 6.0 to 10.0 μL, the DPV peak current of BPA increased first and then decreased, reaching the maximum value at 8.0. With the increase in the volume of CB/f-CNF dispersion, there are more and more composite materials on the surface of GCE, resulting in a gradual increase in peak current, but more and more materials form a thicker film-forming thickness, which will suppress the magnitude of the current. Accordingly, 8.0 μL CB/f-CNF suspension was selected for the following experiment.

BPA has two phenolic hydroxyl groups, and the hydrogen ions in the phenolic hydroxyl group can be deprotonated to form anions at the appropriate pH, which helps to migrate within the electric field. In this experiment, CV was used to study the electrochemical performance of BPA solution with a pH value of 50 μmol L^−1^ in the range of 5.0–9.0. As shown in Figure S4A, the response peak current of BPA gradually increases as the pH increases, indicating that protons are involved in the reaction process between CB/f-CNF and bisphenol. The peak BPA current then gradually decreases at a higher pH, which may be caused by the electrostatic repulsion of the negative charge on the surface of the CB/f-CNF electrode with anionic bisphenols. When the pH value is 7, the oxidation peak current of BPA is at its maximum. At this time, the pH is less than the pKa (9.73) of BPA, which mainly exists in the form of molecules and is easily adsorbed on the electrode surface. Therefore, a BPA buffer solution with pH = 7.0 is optimal. The linear relationship between the peak potential of BPA and the pH was Ep = −0.0631pH + 0.9885 (R^2^ = 0.9991) and the slope was close to the theoretical value of 59.0 mV/pH (Figure S4B). The result indicated the oxidation reaction of BPA included an equal number of electrons and protons. The reaction mechanism of BPA is shown as follows:


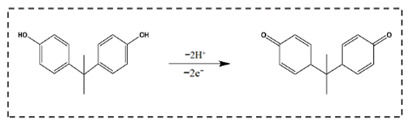


### 3.5. Electrochemical Sensing of BPA on the CB/f-CNF/GCE Sensor

**Figure 5:**
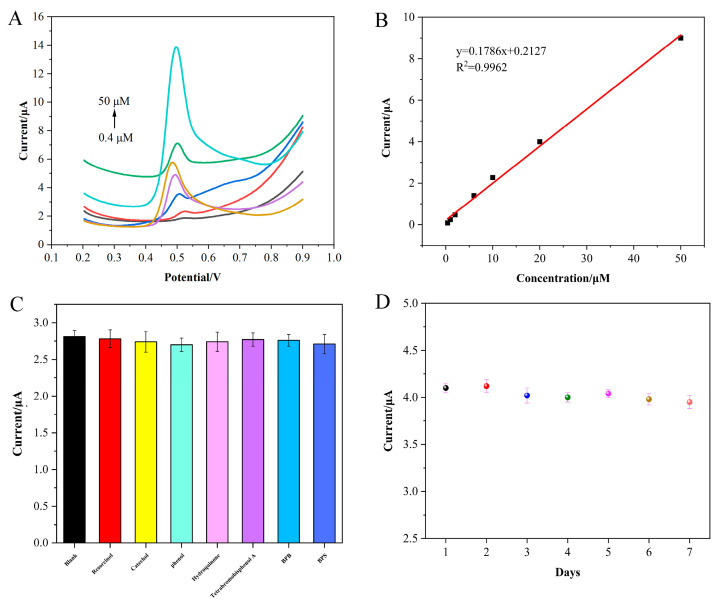
(**A**) DPV curves of CB/f-CNF/GCE for different concentrations of BPA. (**B**) The linear relationship between the peak current and the concentration of BPA was studied, along with anti-interference performance (**C**) and (**D**) repeatability experiments of CB/f-CNF/GCE. Note: In the Figure 5A, the DPV curves of CB/f-CNF/GCE in bisphenol A solution of 0.4 μM, 1 μM, 2 μM, 6 μM, 10 μM, 20 μM and 50 μM are shown in black lines, red lines, blue lines, purple lines, yellow lines, green lines and cyan lines respectively.

The current response of BPA solutions in different concentrations were measured on the CB/f-CNF/GCE sensor. Figure 5A showed the DPV curves of different BPA solution under the optimal conditions. The peak current of BPA increased gradually with the BPA concentrations. The linear equation between the DPV current and the concentration of BPA was I_p_ (μA) = 0.1786C (μM) + 0.2127 with R^2^ = 0.9962. And the limit of detection (LOD) was 0.059 μmol L^−1^. The lower detection limit can be attributed to the enhanced charge transport and the significantly increased surface area of the CB/f-CNF composite. These results demonstrate that CB/f-CNF/GCE is a promising platform for the detection of BPA. A comparison between determinations of BPA using different sensors was investigated and is shown in Table 1. The results clearly demonstrate that CB/f-CNF sensors exhibit excellent sensing capabilities, offering a broader linear range and lower detection limits compared to other modified electrodes.

In the second graph, the dots in different colors represent the CB/f-CNF/GCE stability experiment performed every other day.

### 3.6. The Repeatability, Reproducibility, Stability, and Anti-Interference of the CB/f-CNF/GCE Sensor

CB/f-CNF/GCE prepared in the same manner was used to determine the same BPA (20 μmol L^−1^) solution to assess the repeatability of the constructed sensor. The relative standard deviation (RSD) of the electrodes was 3.96%, suggesting the outstanding repeatability of the CB/f-CNF/GCE sensor. To evaluate the repeatability of the CB/f-CNF/GCE sensor, a single modified electrode was applied for 10 measurements under the same conditions in a 10 μmol L^−1^ BPA solution. The results exhibited that the RSD of the DPV peak currents was 1.96%, indicating the excellent repeatability of the CB/f-CNF/GCE sensor. The stability of the CB/f-CNF sensor for the detection of BPA was also measured in 20 μmol L^−1^ BPA solution. After the CB/f-CNF/GCE was stored for 7 days, the peak current was changed from 4.10 μA to 3.95 μA (96.34% of the original value was reserved), which illustrated the good stability of the CB/f-CNF/GCE sensor.

Anti-interference experiments were conducted by introducing various substances into a 10 μmol L^−1^ BPA solution (Figure 5C), including resorcinol, hydroquinone, catechol, phenol, and tetrabromobisphenol A, as well as bisphenol B and bisphenol F at 20-fold concentrations. When compared to BPA detection in the absence of interferents, the peak current exhibited minimal change. These results demonstrate that the CB/f-CNF/GCE sensor possesses excellent anti-interference properties.

### 3.7. Accuracy and Applicability of the CB/f-CNF Sensor

The recovery experiments were carried out in canned peach in glass jars and milk in paper cartons to confirm the accuracy of the developed sensor. As shown in Table 2, recoveries were in the range of 86.0–102.6% with the RSD of 2.3–4.4%, demonstrating its satisfactory accuracy in real samples.

To evaluate the practical applicability of the CB/f-CNF sensor, the concentrations of BPA in canned yellow peaches in plastic boxes, canned dace with black bean in metal cans, and milk in plastic bags were determined by the proposed sensor and the HPLC method. The detection conditions for HPLC are provided in the Supplementary Materials, and the results are shown in Table S1. The content of BPA in boxed yellow peaches, canned dace with black bean, and bagged milk detected by the CB/f-CNF sensor were 30.1 ± 0.1 μg kg^−1^, 48.4 ± 0.3 μg kg^−1^, and 23.6 ± 0.1 μg kg^−1^, respectively. And those obtained by the HPLC method were 30.8 ± 0.2 μg kg^−1^, 47.8 ± 0.3 μg kg^−1^, and 23.2 ± 0.1 μg kg^−1^, respectively. The results obtained by these two different analytical methods have no significant difference (* *p* > 0.05), indicating that the CB/f-CNF/GCE sensor can be used for the routine determination of BPA in real samples.

## 4. Conclusions

In this work, an electrochemical sensing platform based on CB/f-CNF for signal amplification was developed. The CB/f-CNF/GCE sensor demonstrated a wide linear range (0.4~50.0 × 10^−6^ mol L^−1^), a low LOD of 5.9 × 10^−8^ mol L^−1^, and an LOQ of 2.5 × 10^−7^ mol L^−1^ for BPA detection. The sensor exhibited high accuracy in the quantitative analysis of BPA in real samples including canned food and packaged milk. This study presents a green and environmentally friendly approach, utilizing metal-free carbonaceous nanomaterials to enhance the electrochemical sensing for small-molecule contaminants in food and the environment.

## Figures and Tables

**Table 1:** Comparison between analytical parameters of the proposed sensor and other sensors for the determination of BPA.

Sensor	Linear Range (μM)	LOD (μM)	EC Analytical Methods	Ref.
Tyr-MWCNT/CPE	1–16	1.0	Amperometry	[45]
Laccase-thionine-CB/GCE	0.5–50	0.2	Amperometry	[46]
AuNPs@TpBD-COFs/GCE	5–1000	1.0	DPV	[47]
PDMS@SNCM/ITO	1.0–20.0	0.23	DPV	[48]
CB/f-CNF/GCE	0.4–50	0.059	DPV	This work

**Table 2:** Recovery results of the determination of BPA in samples by the CB/f-CNF/GCE sensor.

Samples	Added Level (μg kg^−1^)	Original Level (μg kg^−1^)	Found Level (μg kg^−1^)	Recovery (%) (mean ± SD, *n* = 3)
Canned peach in glass jars	10	29.8	38.4	86.0 ± 2.3
	30	29.8	60.6	102.7 ± 3.9
	50	29.8	79.3	99.0 ± 3.4
Milk in paper cartons	10	22.4	32.6	102.0 ± 3.6
	30	22.4	52.8	101.3 ± 2.7
	50	22.4	72.1	99.4 ± 4.4

## Data Availability

The original contributions presented in this study are included in the article/Supplementary Material. Further inquiries can be directed to the corresponding authors.

## Supplementary Materials

The following supporting information can be downloaded at: https://www.mdpi.com/article/10.3390/foods14020314/s1, Figure S1. TEM images of CB/f-CNF. Figure S2. Corresponding calibration curves of CV responses in a 2.0 mmol L^−1^ [Fe(CN)_6_]^3/4^- solution at different scan rate from 10 to 100 mV s^−1^. Figure S3. (A) Effect of the volume of the CB/f-CNF dispersion on the peak current. (B) Effect of the the pH of the electrolyte solution on the peak current. Figure S4. (A) CV curves of CB/f-CNF/GCE in 50 μmol/L BPA prepared by different pH of BR. (B) The linear relationship between the response potential and pH value. Table S1. The amount of BPA in actual samples was detected by this method and high performance liquid chromatography.
