# Supplementary material for: A Facile Electrode Modification Approach Based on Metal-Free Carbonaceous Carbon Black/Carbon Nanofibers for Electrochemical Sensing of Bisphenol A in Food
Source: Foods. 2025 Jan 18;14(2):314. doi: 10.3390/foods14020314 (PMC11765411; doi:10.3390/foods14020314)
Supplement: Supplementary file 1 [file foods-14-00314-s001.zip › foods-3416449-supplementary.pdf]

# Supplementary Materials

## A facile synthesis of metal-free carbonaceous nanocomposite (carbon black/carbon nanofibers) for electrochemical sensing of bisphenol A in Food

**Jin Wang[1], Zhen Yang[1], Shuanghuan Gu[1], Mingfei Pan[2,*], Longhua Xu[1,*]**

1 College of Food Science and Engineering, Shandong Agricultural University, Tai'an, Shandong 271018, P.R. China

2 College of Food Science and Engineering, Tianjin University of Science and Technology, Tian'jin, 300457, P.R. China

* Correspondence: longhuaxu@sdau.edu.cn (L. H. Xu); panmf2012@tust.edu.cn (M. F. Pan)

## HPLC validation

High-performance liquid chromatography (HPLC) analysis was carried out using a Shimadzu liquid chromatograph (Japan) equipped with an FL detector. All separations were conducted on a GL Sciences C18 column (250 × 4.6 mm, 5 µm). The excitation wavelength for the FL detector was 227 nm for BPA. The mobile phase was composed of methanol and water (70:30, v/v) with a flow rate of 1.0 mL $min^{-1}$, and the sample injection volume was 15 µL.

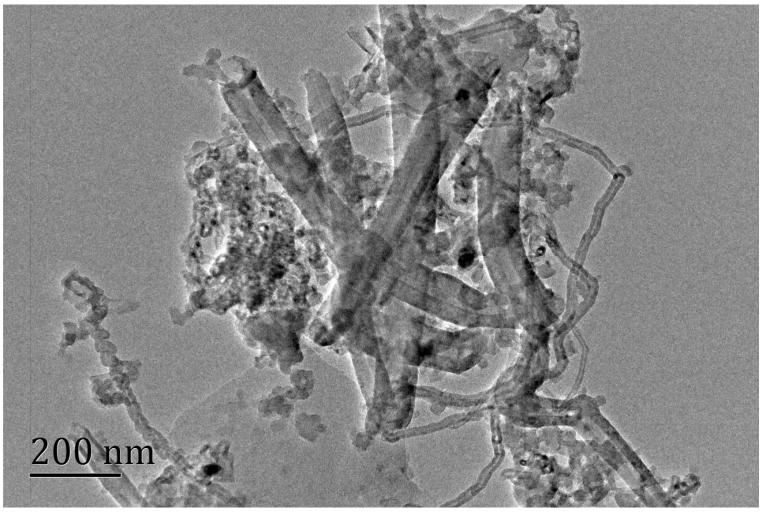


**Fig.S1** TEM images of CB/f-CNF.

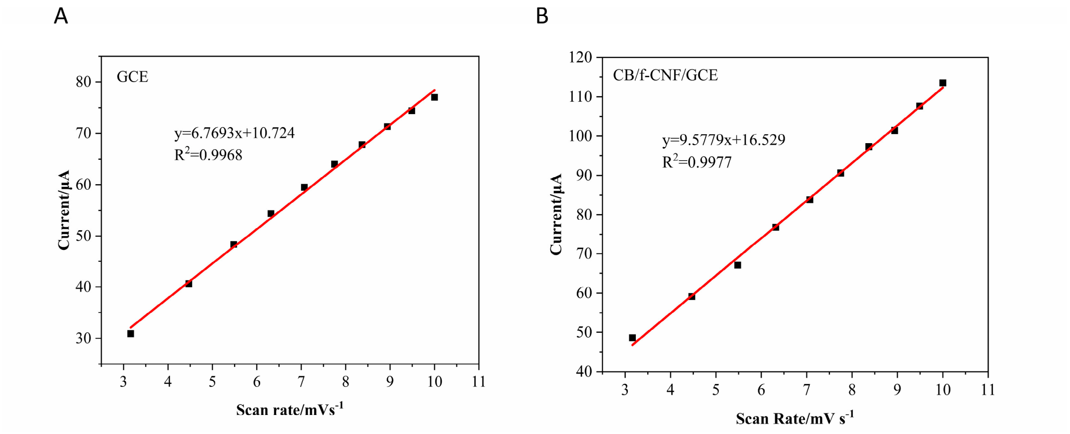


**Fig.S2** Corresponding calibration curves of CV responses in a 2.0 mmol L-1 $[Fe(CN)_6]^{3/4-}$ solution at different scan rate from 10 to 100 mV $s^{-1}$.

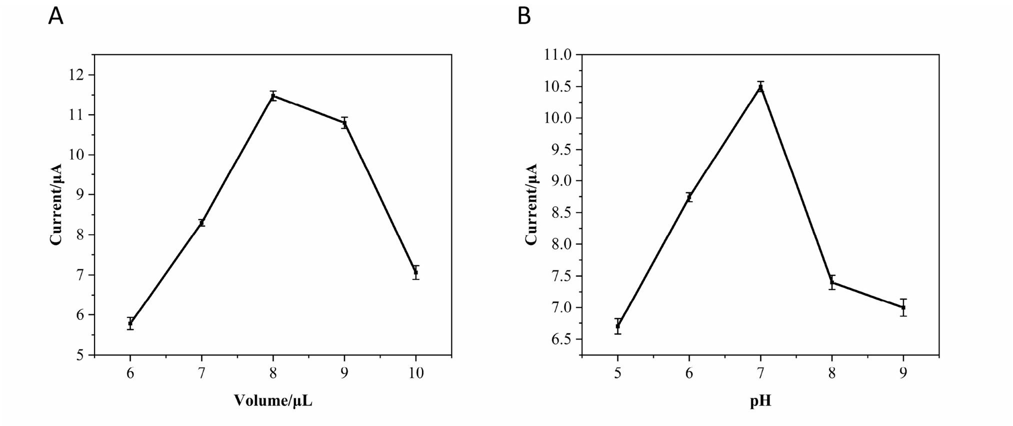


**Fig.S3** (A) Effect of the volume of the CB/f-CNF dispersion on the peak current.(B) Effect of the the pH of the electrolyte solution on the peak current

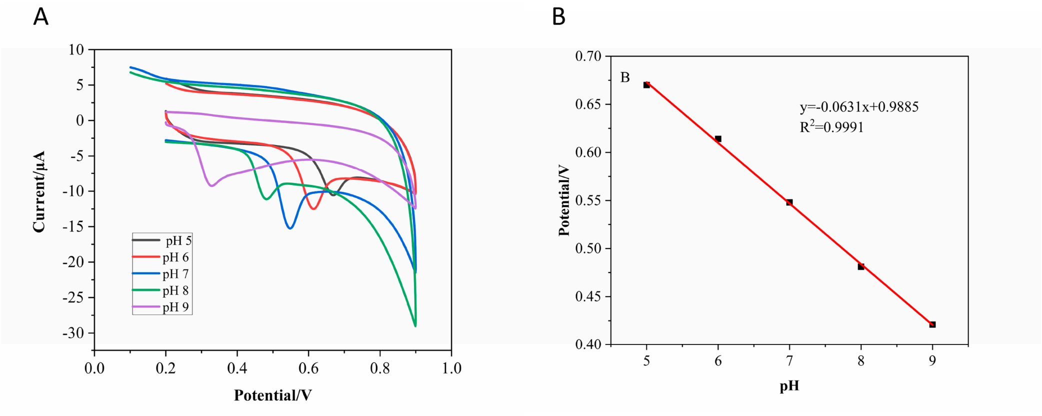


**Fig.S4** (A) CV curves of CB/f-CNF/GCE in 50 μmol/L BPA prepared by different pH of BR.(B) The linear relationship between the response potential and pH value.

**Table S1** The amount of BPA in actual samples was detected by this method and high performance liquid chromatography

| Samples | Found level by the sensor (μg kg$^{-1}$, ± SD) | Found level by HPLC (μg kg$^{-1}$, ± SD) | P* |
|---|---|---|---|
| canned yellow peach in plastic boxes | 30.1 ± 0.1 | 30.8 ± 0.2 | 0.27 |
| canned dace with black bean in metal cans | 48.4 ± 0.3 | 47.8 ± 0.3 | 0.12 |
| milk in plastic bags | 23.6 ± 0.1 | 23.2 ± 0.1 | 0.07 |
